# The relationship between BMI and the prescription of anti-obesity medication according to social factors: a population cross sectional study

**DOI:** 10.1186/1471-2458-14-87

**Published:** 2014-01-28

**Authors:** Lynsey Patterson, Frank Kee, Carmel Hughes, Dermot O’Reilly

**Affiliations:** 1 UKCRC Centre of Excellence for Public Health (Northern Ireland), Queen’s University Belfast, Belfast BT12 6BJ, Northern Ireland; 2 Centre for Public Health, Queen’s University Belfast, Belfast BT12 6BJ, Northern Ireland; 3 School of Pharmacy, Queen’s University Belfast, Belfast BT9 7BL, Northern Ireland

**Keywords:** General practice, Anti-obesity agents, Socioeconomic factors, Weight loss, Obesity, Multi-level modelling

## Abstract

**Background:**

Obesity is a global public health problem. There are a range of treatments available with varying short and long term success rates. One option is the use of anti-obesity medication the prescription of which has increased dramatically in recent years. Despite this, little is known about the individual and GP practice factors that influence the prescription of anti-obesity medication.

**Methods:**

Multi-level logistic regression analysis was used to investigate factors associated with the prescription of anti-obesity medication in Northern Ireland using a population primary care prescribing database (~1.5 million people aged 16+ years) during 2009/10.

**Results:**

While 25.0% of people are obese, only 1.3% (2.1% of females, 0.6% of males) received anti-obesity medication. The relationship between medication rates and age differed by gender (P < 0.001) with prescriptions higher in younger females and older males. Prescribing of anti-obesity medication reflected obesity prevalence across urban/rural areas and deprivation. There was an unexplained two-fold difference, between the 25^th^ and 75^th^ percentile, in the GP practice prescription of anti-obesity medication.

**Conclusions:**

There is evidence of relative under-prescribing in males compared to females despite a similar prevalence of obesity. While the prevalence (and presumably the health consequences) of obesity worsens with age, younger females are more likely to be prescribed anti-obesity medication. This suggests an element of patient demand. Educational material to improve the understanding of the role of anti-obesity medication, for patients and practitioners, is recommended. But further study is needed to understand the factors responsible for the variation in prescribing between GP practices.

## Background

In the UK, it is estimated that up to 40% of the population could be obese by 2030, based on recent trends [1]. This equates to an estimated £1.9 billion excess spend in terms of healthcare costs [1]. The main interventions for weight loss, behaviour and lifestyle changes, are difficult to achieve and have modest long-term success rates [2]. As the prevalence of obesity rises, pressure to find an appropriate intervention, such as bariatric surgery, increases [3]. Whilst there would be huge economic benefits if those eligible for surgery received it, [4] only a small proportion of the population meet the current threshold for this intervention (Body Mass Index ≥ 40 kg/m^2^).

In recent years, pharmacological treatment for obesity has been increasing against a backdrop of decreasing choice of available anti-obesity medications. Orlistat (brand names Xenical/Alli) was first introduced in the UK and Europe in 1998 and is currently the main treatment option for those prescribed anti-obesity medication. In 1999, sibutramine (brand name Reductil) was introduced as an anti-obesity treatment option but was subsequently removed in January 2010 because of the risk of cardiovascular disease [5]. In 2006, Rimonabant (brand name Acomplia) was marketed as an anti-obesity drug but was removed in November 2008 because of the risk of psychiatric side effects [6].

In Northern Ireland, during 2009/2010 orlistat accounted for approximately 80% of prescriptions for anti-obesity medication increasing to 99% following the withdrawal of sibutramine. In a meta-analysis of randomised control trials (RCTs) orlistat in conjunction with a weight loss diet reduced weight by 2.9 kg (95% CI 2.5 kg – 3.2 kg) and slightly increased the proportion of patients achieving 5% and 10% weight reduction thresholds [7]. It is not clear what proportion of this reduction is due to the effects of diet versus medication use but a reduction of this magnitude, if achieved, is sufficient to significantly decrease mortality and the risk of developing cardiovascular disease or cancer [8]. Some studies have shown that anti-obesity medications are a cost-effective form of treatment [9,10], however, the research is often funded by pharmaceutical companies and relies on large assumptions [11]. Despite the potential benefits of using anti-obesity medication a history of safety fears and a variety of side effects may have hampered their use [5,12]. The latter has been observed in RCTs of orlistat treatment where attrition rates of 30% were observed across a range of RCTs [7].

Being overweight and obese is associated with increasing age [13], gender (higher in males) [14], increasing deprivation [15] and the built environment [16]. It is therefore conceivable that the same factors may be associated with the likelihood of being prescribed an anti-obesity medication. Variations may also exist at a general practice level reflecting the beliefs and practices of primary care physicians [17].

This study aims to: 1) relate uptake of anti-obesity medications to need and; 2) quantify the amount of variation in prescribing of anti-obesity medication between general practices.

## Methods

This was a cross sectional analysis of anti-obesity medication prescribing in the Northern Ireland (NI) population (~1.5 million people aged 16+ years). Data was combined from two different sources to relate obesity levels for different socio-demographic groups to the prescription of anti-obesity medication among these groups.

Physical measurements of height and weight from the NI Health and Wellbeing Survey (NI HWBS 2005/06) were used to produce population estimates of obesity (BMI ≥ 30 kg/m^2^) for the population aged 16 years and over. The 2005/06 survey year was chosen as the most contemporaneous data on objectively measured height and weight for a representative sample of the NI population. Data on all medications prescribed by GPs and dispensed by community pharmacists are collated centrally in an enhanced prescription database (EPD) and in 2009 the population coverage was approximately 80% (the shortfall being due to software incompatibility, but the 80% are known to be representative of the population in terms of demography and geographical spread). Prescribing data were extracted for the period 1^st^ April 2009 – 31^st^ March 2010, to reflect the most recent data available.

During 2009/10, approximately 85% of the population had access to free prescriptions. For those that had to cover the cost of their prescription, mainly those from the most affluent groups, there was a minimal fee of approximately £3 per prescription. Individuals were excluded from the analysis if they had died, emigrated, were institutionalised or were under 15 years of age during the study period (n = 412,795). After exclusions, the prescribing data related to a population of 1,492,982 individuals. The primary outcome was the prescription of anti-obesity medication (British National Formulary category 4.5 (orlistat, sibutramine and rimonabant)/ATC codes A08AB01 (orlistat), A08AA10 (sibutramine), and A08AX01 (rimonabant).

Predictors included age, classified into seven age groups (Nine year age bands from 16-24, through to 75 and over) and gender. An ecologically based indicator of socioeconomic status and of urban/rural residence was also included. The former was based on the uptake of means tested benefits and derived from the 2010 NI multiple deprivation measure (NIMDM; http://www.nisra.gov.uk). It was calculated at the Super Output Area with an average population of ~2000 people. The distinction between urban and rural dwelling individuals used the “settlement band” classification (http://www.nisra.gov.uk) which is based on population size, population density and service provision. The settlements were grouped as urban (band A, comprising the largest city and hinterland), intermediate (bands B-G) or rural dwelling (band H). General practice variables included GP median age (grouped), gender ratio, practice population size and the practice prevalence of obesity, from the Quality and Outcomes Framework (QOF), categorised into deciles (one = lowest; 10 = highest prevalence). The study was approved by the Office for Research Ethics Committee (ORECNI; REC Reference number 10/NIR02/19).

### Analytical strategy

The analysis was divided into two parts; the first utilised multi-level logistic regression models to describe the characteristics of patients who were prescribed anti-obesity medication, adjusting for the clustering of patients within practices. Each of the factors described were included in univariate analysis and factors significantly associated with the likelihood of being prescribed anti-obesity medication (P < 0.05) were included in the final adjusted model (age group, settlement band and income). Interaction tests were carried out to determine if there were significant differences according to gender. The Variance Partition Coefficient (VPC) was calculated using a linear threshold model [18]. The VPC describes the importance of the general practice in explaining the overall variation in the prescription of anti-obesity medication. The median Odds Ratio (mOR) was also calculated to quantify the variation between clusters by comparing two identical individuals from two randomly chosen different clusters [19]. The MOR can be directly compared to the odds ratio for individual level variables [18].

The second part of the analysis related the distribution of anti-obesity medication prescriptions to the estimated number of obese individuals in the population derived from the Health Survey to estimate the proportion of obese individuals that received anti-obesity medication. All analysis was carried out in STATA version 10 (STATA Corp., College Station, TX, USA).

## Results

### Socio-demographic analysis of prescribing

During the study year an estimated 1.3% of the population received anti-obesity medication; the prevalence was higher among women (2.1%) than men (0.6%). The gender difference in the prescription of anti-obesity medication, which was higher in females, was evident at all ages; prescribing in females peaked at younger ages and to a greater extent than in men (Table 1). Approximately 3.0% of women aged 35–54 received anti-obesity medication, compared to less than half that proportion in men aged 45-64 years (the age of greatest prescriptions for men). It is notable that amongst women the prescription of anti-obesity medication was significantly higher at younger (35–54 years) than at older ages, but the reverse was true for men. For both males and females, the proportion of the population prescribed anti-obesity medication fell significantly over the age of 65. A significant interaction test in the logistic regression confirmed that the prescription of anti-obesity medication by age varied according to gender (LR *χ*^2^_(6)_ = 437.7; P < 0.001), so all subsequent analyses were stratified by gender.

**Table 1:** The socio-demographic factors associated with the prescription of an anti-obesity medication and measures of clustering at the practice level

	**Women**			**Men**		
	**Total population (% on medication)**	**OR**^**1**^**(95% CI)**	**P-value**	**Total population (% on medication)**	**OR**^**1**^**(95% CI)**	**P-value**
**Age group**						
16-24	127 719 (0.9)	0.29 (0.27, 0.31)	<0.001	133 610 (0.1)	0.15 (0.13, 0.18)	<0.001
25-34	132 355 (2.2)	0.71 (0.68, 0.75)	<0.001	135 349 (0.4)	0.49 (0.44, 0.55)	<0.001
35-44	136 022 (3.0)	1.00		141 154 (0.7)	1.00	
45-54	123 844 (2.9)	0.98 (0.93, 1.02)	0.35	127 073 (0.9)	1.29 (1.19, 1.40)	<0.001
55-64	96 110 (2.6)	0.88 (0.84, 0.93)	<0.001	96 422 (1.0)	1.42 (1.30, 1.55)	<0.001
65-74	72 726 (1.5)	0.50 (0.46, 0.53)	<0.001	65 679 (0.7)	0.98 (0.88, 1.09)	0.71
75+	61 728 (0.3)	0.09 (0.08, 0.10)	<0.001	39 721 (0.2)	0.22 (0.17, 0.28)	<0.001
**Settlement band**						
Urban	290 578 (2.5)	1.00		278 807 (0.7)	1.00	
Intermediate	248 335 (2.0)	0.97 (0.89, 1.05)	0.43	239 862 (0.6)	0.90 (0.80, 1.01)	0.065
Rural	199 655 (1.6)	0.86 (0.79, 0.93)	0.001	208 669 (0.5)	0.81 (0.72, 0.91)	<0.001
**Area deprivation**						
Least deprived	154 850 (1.6)	1.00		149 806 (0.5)	1.00	
2^nd^	162 180 (1.8)	1.22 (1.15, 1.30)	<0.001	159 870 (0.6)	1.19 (1.08, 1.32)	0.001
3^rd^	131 458 (2.0)	1.45 (1.36, 1.54)	<0.001	129 325 (0.5)	1.16 (1.04, 1.30)	0.009
4^th^	149 087 (2.2)	1.64 (1.55, 1.74)	<0.001	146 440 (0.6)	1.28 (1.15, 1.42)	<0.001
Most deprived	140 993 (2.9)	1.95 (1.83, 2.07)	<0.001	141 897 (0.8)	1.64 (1.47, 1.82)	<0.001
**Measures of variation or clustering**						
Practice level variance (SE)	0.25 (0.024)			0.21 (0.026)		
Variance Partition Coefficient (%)	7.2			6.1		
Median Odds Ratio	1.61			1.55		

^1^Odd Ratio (OR) and 95% Confidence Intervals from fully adjusted multi-level logistic regression models stratified by gender.

The prescription of anti-obesity medication was significantly higher in urban than in rural settings, although in the fully adjusted models the gradients were more apparent for men than for women. Compared to individuals in urban areas, men in rural areas were 19.0% less likely to receive medication (95% CI for OR: 0.72, 0.91; P < 0.001) and women in rural areas were 14.0% less likely to receive anti-obesity medication (95% CI for OR: 0.79, 0.93; P = 0.001).

There was a steep and graded significant relationship between the prescription of anti-obesity medication and deprivation in females with a twofold higher likelihood of receiving anti-obesity medication in the most deprived compared to the least deprived areas (OR 1.95 95% CI: 1.83, 2.07; P < 0.001 (fully adjusted model)). The relationship with deprivation was less marked in males, with the most deprived being 64.0% more likely to be prescribed medication than those in the least deprived quintile (95% CI for OR: 1.47, 1.82; P < 0.001).

### Practice variation

The proportion of the general practice population that was prescribed an anti-obesity medication ranged from 0% - 4.5% across the 358 practices, with an approximate twofold difference between the 25^th^ and 75^th^ percentile. The proportion of variation in prescribing that can be attributed to the general practice was 7.2% for females and 6.1% for males (VPC estimates). On the odds ratio scale this translates to a median odds ratio of 1.61 in females and 1.55 in males. There was no association found between the prevalence of obesity amongst the practice population, the practice population size or the median age/gender ratio of the GPs and the likelihood of being prescribed anti-obesity medication.

### Prescribing versus need

Table 2 shows how the estimated prevalence of obesity from the Health and Wellbeing Survey varies across the socio-demographic factors described above and relates the prescription of anti-obesity medication to these estimates. Overall, 24.4% of males and 23.5% of females were estimated to be obese. However, fewer obese males than obese females were prescribed anti-obesity medication (2.4% in males compared to 8.8% in females).

**Table 2:** The proportion of obese individuals in the population and the estimated proportion of obese men and women receiving anti-obesity medication according to age and area of residence

		**Women**			**Men**	
	**Obesity Prevalence**	**Patients on anti-obesity medication**	**Estimated % obese patients on treatment**	**Obesity prevalence**	**Patients on anti-obesity medication**	**Estimated % obese patients on treatment**
**Age group**						
16-24	13.6	1162	6.7	12.0	152	0.9
25-34	23.7	2893	9.2	23.5	495	1.6
35-44	27.2	4070	11.0	28.5	1046	2.6
45-54	26.6	3641	11.1	31.9	1198	3.0
55-64	28.2	2511	9.3	29.3	982	3.5
65-74	25.6	1115	6.0	27.2	472	2.6
75+	19.8	173	1.4	15.4	64	1.0
**Settlement band**						
Urban	22.9	7111	10.7	18.8	1978	3.8
Intermediate	26.5	5009	7.6	28.9	1375	2.0
Rural	20.6	3222	7.8	25.6	994	1.9
**Area deprivation**						
Least deprived	19.8	2488	8.1	19.3	778	2.7
2^nd^	21.3	2856	8.3	27.0	896	2.1
3^rd^	23.7	2605	8.4	27.7	670	1.9
4^th^	26.1	3302	8.5	24.7	864	2.4
Most deprived	28.4	4091	10.2	23.8	1139	3.4

Comparing the prevalence of obesity to the number of people prescribed anti-obesity medication we estimated that one in 10 obese females in the 35–54 year age groups received anti-obesity medication. In all other age groups, the proportion receiving anti-obesity medication was less than this, with 6.0% of obese 65–74 year olds and 1.4% of those over 75 years receiving anti-obesity medication. Compared to females, the estimated proportion of obese males receiving anti-obesity medication was lower across all age groups. Excluding the 16–24 and >75 years age groups where approximately 1.0% of obese males were being treated, an estimated 2.0–3.0% of obese males across all other age groups were prescribed anti-obesity medication (range 1.6% – 3.5%).

Approximately one in 10 obese females in urban areas received anti-obesity medication compared to 7.6% and 7.8% in intermediate and rural areas respectively. For males the proportion of obese individuals receiving anti-obesity medication is much lower; approximately 3.8% of males in urban areas compared to 2.0% in the intermediate band and 1.9% in rural areas.

For both males and females, the socio-economic gradient in estimated prescription of anti-obesity medication matched or exceeded that of obesity prevalence. In females, an estimated one in 10 obese individuals received anti-obesity medication in the most deprived areas compared to 8.1% in the most affluent. For males the difference was smaller with 3.4% of obese individuals receiving ant-obesity medication in the most deprived areas compared to 2.7% in the most affluent.

## Discussion

### Main finding of this study

The data has shown that during this study period only a small proportion of obese individuals are receiving anti-obesity medication (approximately 5.8% overall). Whilst the prevalence of obesity is similar in males and females, females, and particularly those from younger age groups, are more likely to be prescribed anti-obesity medication.

### What is already known on this topic

Anti-obesity medication has been shown, in clinical trials, to be an effective method of weight loss with a consequential reduction in health risks [20]. The increased prescription of anti-obesity medication has been reported both in the UK [21], the US [22] and Canada [12]. The US study, which used the Behavioural Risk Factor Surveillance System across five states, was based on self reported pill use and showed a higher use of anti-obesity medication amongst females [22]. A UK study, based on the prescription cost analysis system, reported a 25-fold rise in orlistat prescriptions between 1998 and 2005 but presented no further information about the individuals receiving the prescriptions or the GP practices prescribing them [21].

### What this study adds

Although it is known that the annual number of prescriptions and cost of prescribed anti-obesity medication has increased there is little information about how prescription of these drugs relates to need and how much it is influenced by the prescriber. To our knowledge, this study is the first to explore both individual (patient) and group (practice) level factors associated with the likelihood of being prescribed anti-obesity medication using multilevel statistical modelling. It also adds to the existing literature by including deprivation and urban/rural factors, which are known to be associated with obesity.

The alignment of prescribing and prevalence data has allowed us to estimate the equity of the prescription of anti-obesity medication and while there are many aspects where prescribing appeared aligned with need, there were examples where it is not. The most obvious is the relative difference between the prescription of anti-obesity medication amongst obese men and women. The health survey data show that there were approximately equal proportions of obese men and women, but at most ages it appeared that women were more than three times as likely to receive anti-obesity medication compared to men. This disparity is even more marked considering the higher prevalence of diabetes in men [23] and their greater risk of cardiovascular disease [24,25]. There are further disconnects between levels of obesity and rates of prescribing across the age spectrum, especially amongst women, with increased prescribing among obese younger females compared to the older age groups. Again, this is not in keeping with the gradients in risk as it is older post-menopausal women who are likely to experience the greatest health gains from weight loss, as the risk of other adverse outcomes, such as cardiovascular disease, increases [26]. Whether the gender and age differences observed reflect a better perception of need and greater health seeking behaviour amongst women or a lack of awareness and reticence in seeking medical care amongst men is not known [27]. Gender differences in the prescription of anti-obesity medication may also reflect the individual’s perception of their body weight and dissatisfaction with their body image. Females are more likely to identify that they need to lose weight even if they fall into the ‘normal’ category, using criteria defined by the World Health Organisation [28]. Whilst women experience body dissatisfaction throughout their lifetime, the impact this has on self-esteem diminishes with age [29] which may account for the fewer prescriptions in the older age group.

The inverse care law, as proposed by Tudor Hart [30], states that “the availability of good medical care tends to vary inversely with the need for it in the population served” [30]. This has been observed for a range of health care services but was not evident in this study [31]. There are two caveats: the first is that the ecologically assigned socioeconomic status assumes that it is the deprived people in the deprived areas who are getting the medication; and secondly, the observed gradient may still represent inequality [32] given the increased risk of negative health outcomes in the more deprived populations [33,34].

The practice effect on the likelihood of being prescribed anti-obesity medication is evident for both males and females. For females, the residual heterogeneity between practices (mOR = 1.61) was comparable to living in the 4^th^ most deprived quintile (OR = 1.64) and was of greater importance than age or settlement band for understanding the variation in the odds of being prescribed anti-obesity medication. In males, the residual heterogeneity between practices (mOR = 1.55) was comparable to the most deprived quintile (OR = 1.64) and was of greater importance than age or settlement band for understanding the variation in the odds of being prescribed anti-obesity medication. The variation in the prescription of anti-obesity medication at the general practice level may be related to reluctance by some General Practitioners (GPs) to prescribe anti-obesity medication if they believe obesity is better treated through lifestyle changes [35]. Indeed, using anti-obesity medication is cited as the least likely treatment approach by physicians [35]. It is also possible that both individuals and GPs avoid the use of pharmacotherapy because of a disconnect in their beliefs about the causes and solutions of obesity [36,37], an uncertainty about appropriateness and safety, concern about side effects particularly for orlistat [36], or because of the availability of alternative management options, for example, access to dieticians.

### Strengths and limitations

This study had two main strengths: 1) the ability to access individual level data for the prescription of anti-obesity medication across the population and; 2) the use of multi-level modelling to account for individual and GP practice factors associated with the prescription of anti-obesity medication. A limitation to this analysis was the disconnect in the time periods used to describe obesity prevalence (2005/06) and the prescribing of anti-obesity medication (2009/10). Whilst the time periods do not overlap it is unlikely that the patterning of obesity prevalence will have changed over this time. It is possible that the overall prevalence of obesity may have changed; the direction for this is likely to have been a slight increase and therefore estimates relating need to observed prescribing are conservative. Furthermore, as this study was observational in nature it was not possible to infer causality. An assumption was made that prescribing should reflect the National Institute for Health and Clinical Excellence (NICE) guidelines. However, we cannot infer the clinical basis for the prescription; that the individuals were obese or were overweight with co-morbid conditions with unsuccessful weight loss after at least three months of managed care. We also cannot confirm that the medication was actually taken nor could we account for patient or practitioners beliefs and knowledge of obesity, in general, or of anti-obesity medication. It is possible, therefore, that variations in prescribing may be due to clinical contraindications or to patient/practitioner preference but the size of the differences observed would make this unlikely. It would have been helpful to have individual-level data relating to BMI as well as other personal attributes such as socio-economic status. These data were not readily available.

Our study lacked a measure of other risk factors which presumably would be higher in males; therefore the male to female ratio of “need versus demand” is likely to be more marked than reported here. The analysis is based on the prescription of anti-obesity medication from GPs, where the majority of prescribing for anti-obesity medication occurs. This excludes drugs dispensed in hospitals, private prescriptions or those supplied over-the-counter by community pharmacies. The latter may be the treatment of choice for those in more affluent populations.

## Conclusions

Overall, the pattern of prescribing for anti-obesity medication appears to reflect the needs of the population. However, there is some evidence of socially determined prescribing, specifically reflected in under-prescribing amongst males and higher prescribing amongst younger females. Further studies linking an individual’s BMI and co-morbidity to the prescription of anti-obesity medication may help to elucidate if individual level need is being met. Educational material to inform the proper use of anti-obesity medication, amongst patients and practitioners, and to help with realistic goal setting should be encouraged. There is also a need to understand the factors responsible for the variation in prescribing between practices.

## Competing interests

The authors declare that they have no competing interests.

## Authors’ contributions

LP, FK, CH and DOR contributed to the planning including the design and interpretation of the data; LP was responsible for the acquisition of the data and the analysis; all authors contributed to the drafting, revising and final approval of the manuscript.

## Pre-publication history

The pre-publication history for this paper can be accessed here:

http://www.biomedcentral.com/1471-2458/14/87/prepub
